# Supplementary material for: Expression-based subtypes define pathologic response to neoadjuvant immune-checkpoint inhibitors in muscle-invasive bladder cancer
Source: Nat Commun. 2023 Apr 27;14:2126. doi: 10.1038/s41467-023-37568-9 (PMC10140274; doi:10.1038/s41467-023-37568-9)
Supplement: Supplementary file 1 — Supplementary Information [file 41467_2023_37568_MOESM1_ESM.pdf]

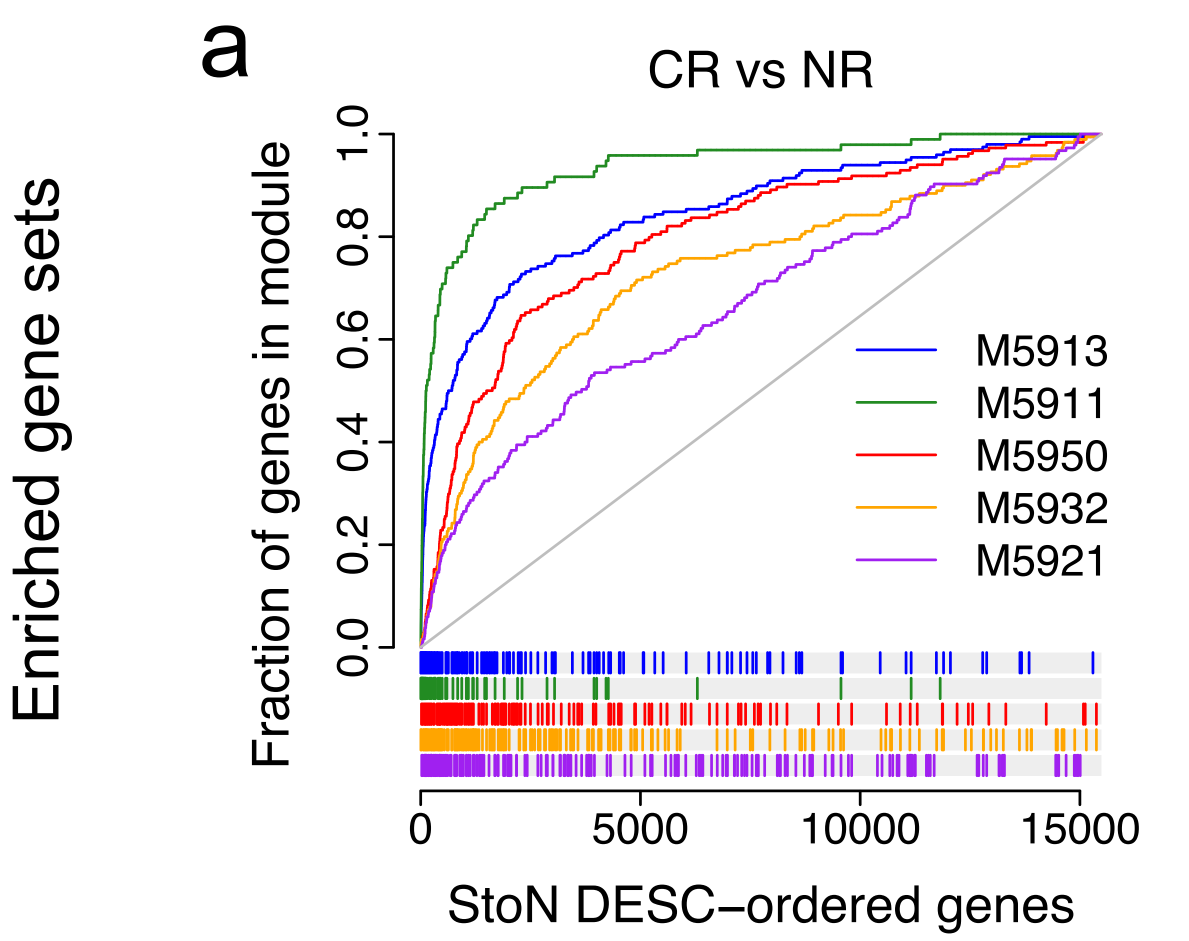

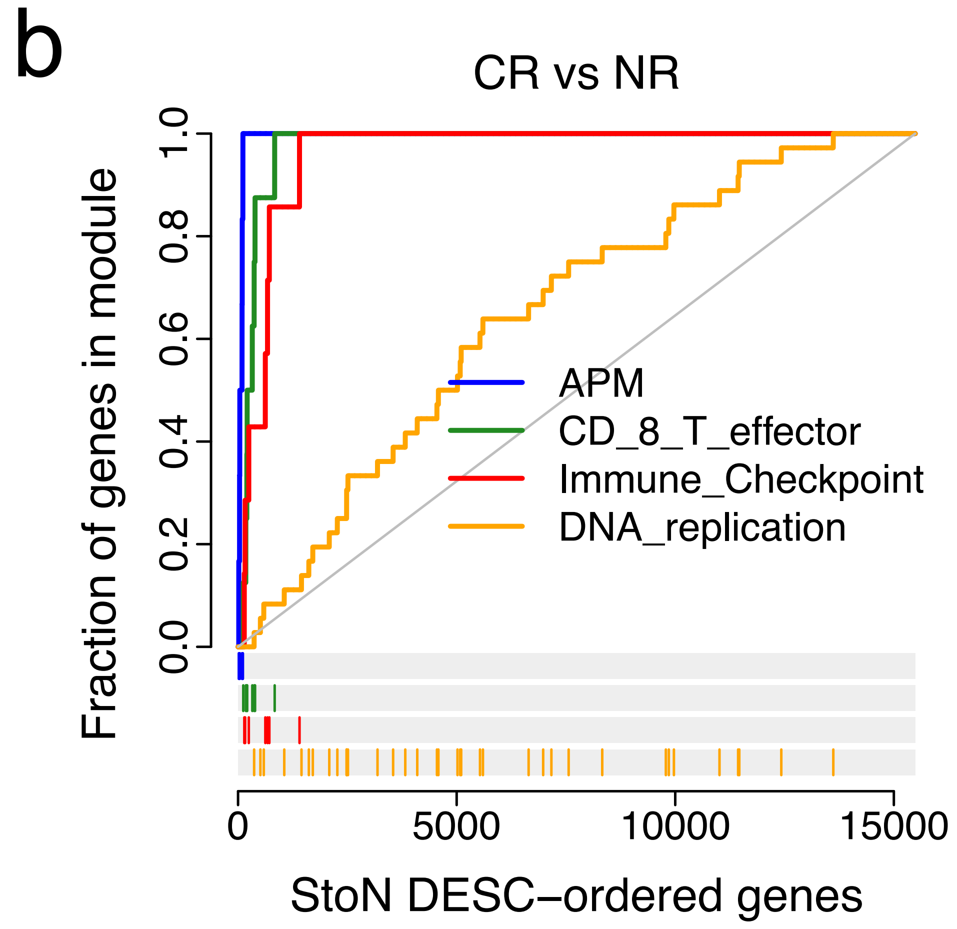

ID	Title	AUC	FDR
M5913	IFNG response	0.85	4.6E-104
M5911	IFNA response	0.94	7.5E-86
M5950	allograft rejection	0.81	8.7E-45
M5932	inflammatory response	0.73	6.2E-32
M5921	complement	0.67	4.0E-19
M5890	tnfa signaling via nfkb	0.67	1.3E-15
M5897	il6 jak stat3 signaling	0.73	2.6E-12
M5902	apoptosis	0.61	3.1E-08
M5947	il2 stat5 signaling	0.61	3.4E-08
M5953	kras signaling up	0.62	1.2E-07
M5938	ROS pathway	0.63	4.6E-03

Title	AUC	FDR
APM	1.00	1.5E-08
CD 8 T effector	0.98	9.5E-07
Immune Checkpoint	0.96	3.5E-05
DNA replication	0.65	0.080

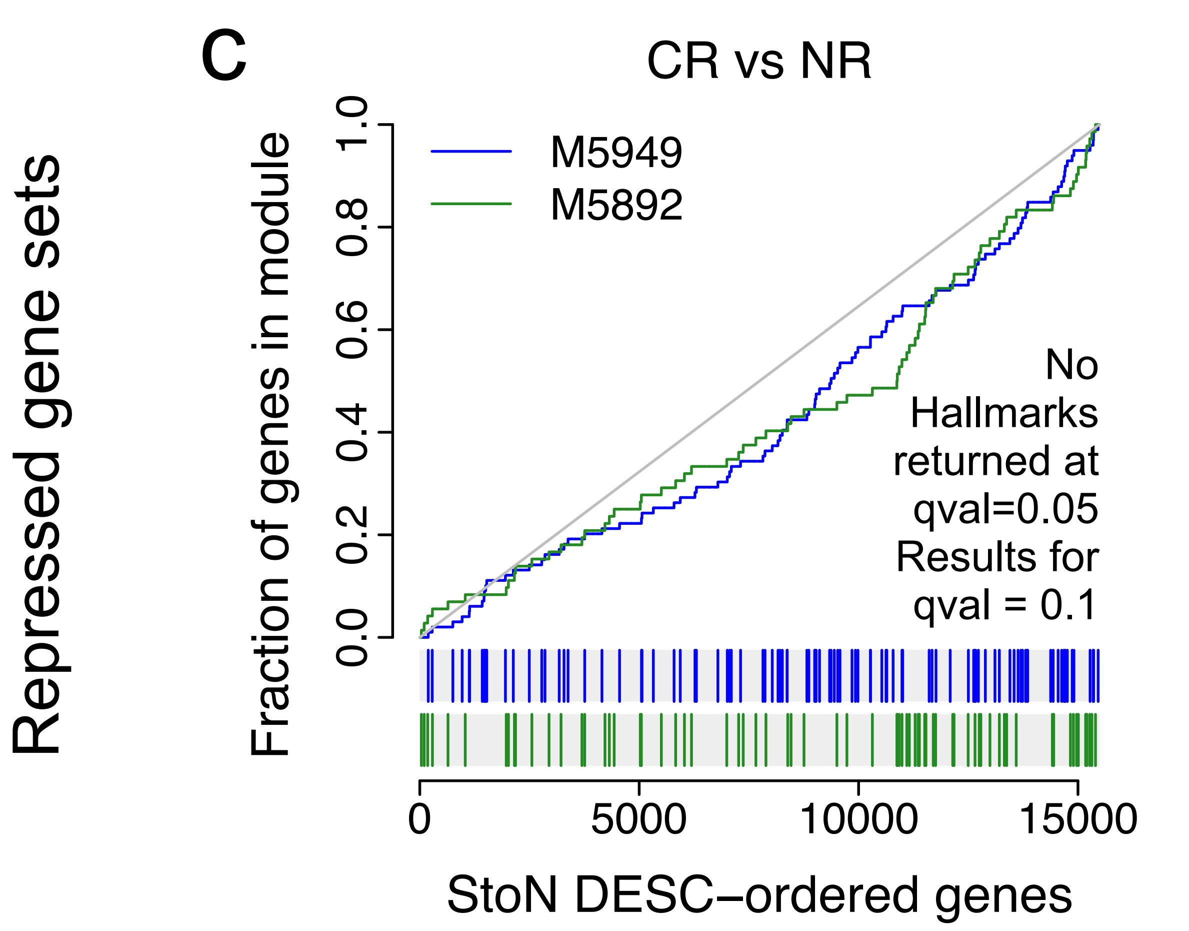

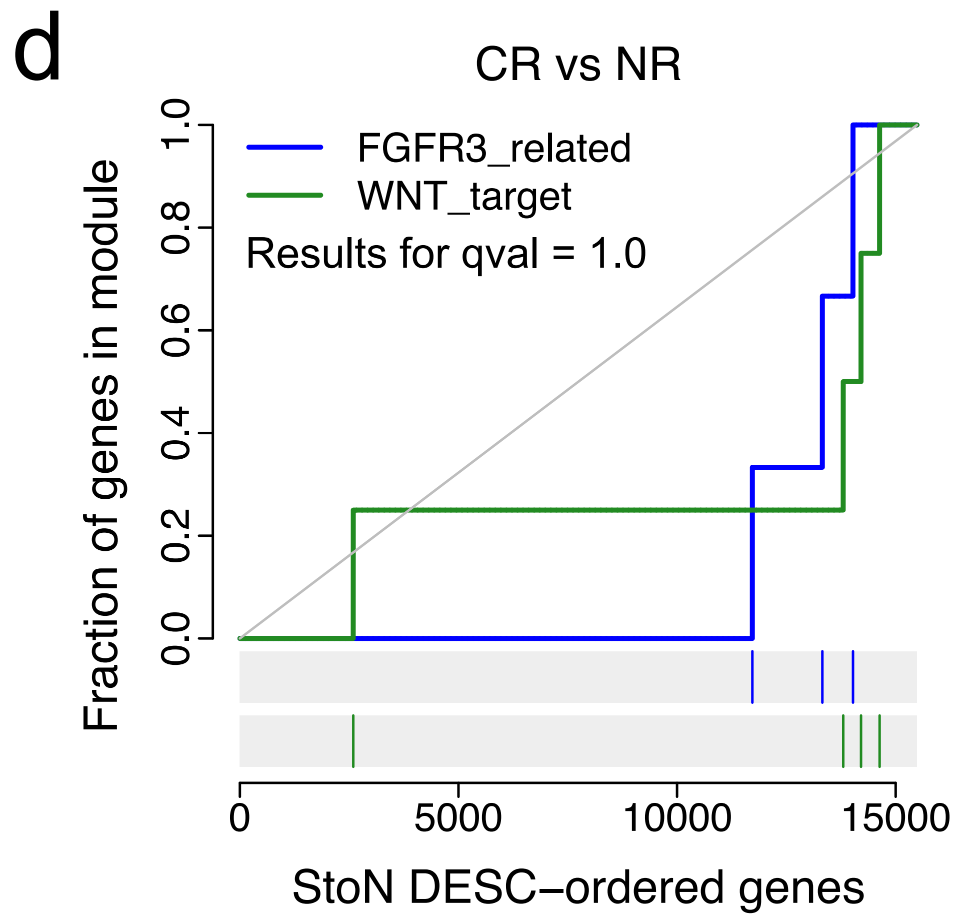

ID	Title	AUC	FDR
M5949	peroxisome	0.58	0.095
M5892	cholesterol homeostasis	0.58	0.095

Title	AUC	FDR
FGFR3-related	0.84	0.746
WNT target	0.73	0.746

Supplementary Figure 1. Enriched and repressed gene sets for PURE01 n=82 CR vs. NR samples.

a) Enriched Hallmarks: enrichment plot (above) and tabular results (below) from CERNO tests on 50 MSigDB v7.2 Hallmark gene sets.

b) As (a), enriched gene sets from twenty Mariathasan[20] gene sets.

c) Repressed Hallmark gene sets.

d) Repressed Mariathasan gene sets.

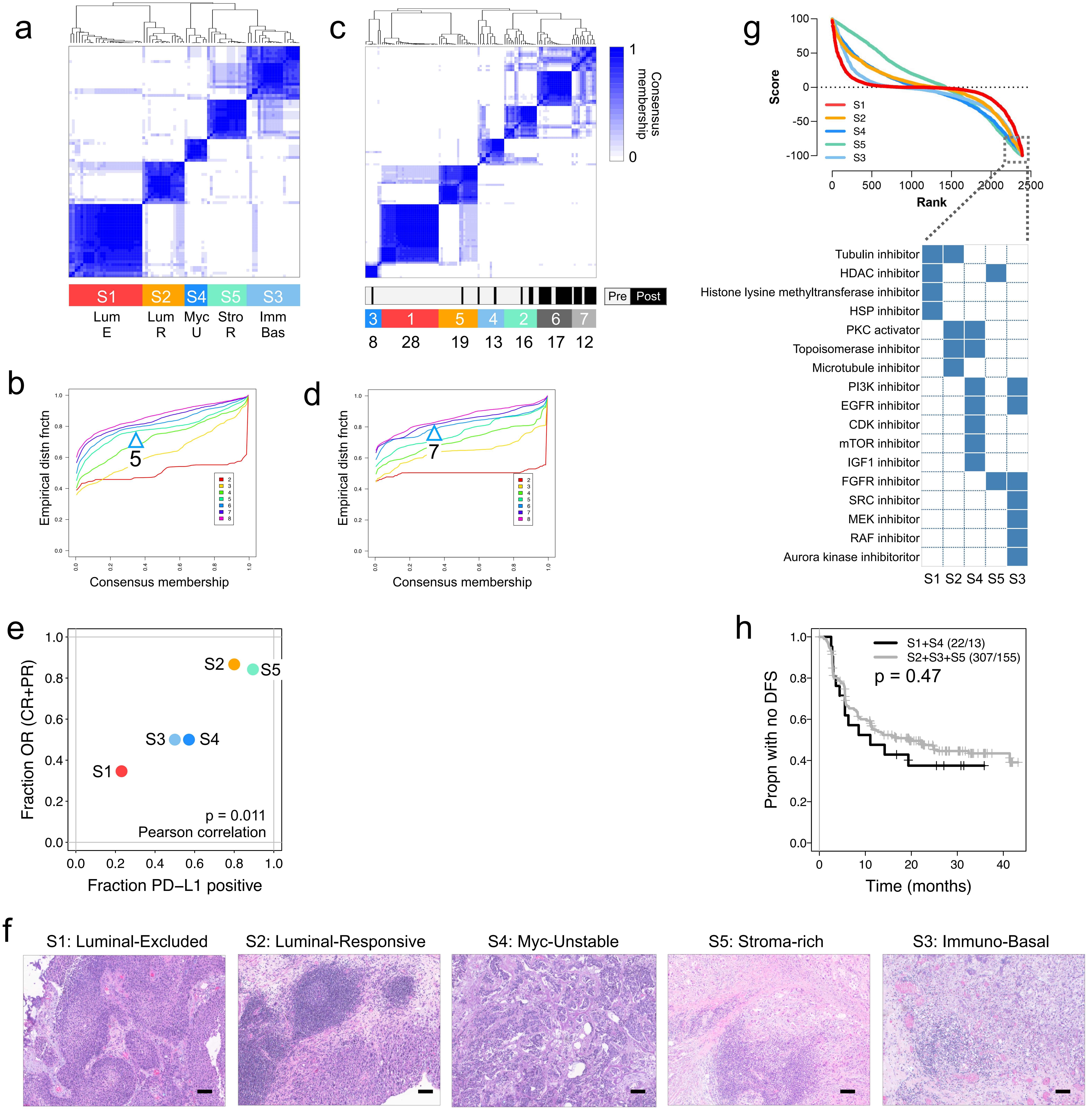

Supplementary Figure 2

Supplementary Figure 2. Consensus clustering for PURE01 n=82 and n=113 cohorts, PD-L1 and Overall Response, and H&E micrographs.

a,b) Consensus membership heatmap for a five-cluster solution for the PURE01 n=82 pre-treatment cohort, with CDF plots from ConsensusClusterPlus.

c,d) As (**a,b**), but for the seven-cluster solution for the PURE01 n=113 pre-and-post-treatment cohort.

e) In each PURE01 n=82 subtype, the relationship between the fraction of PD-L1(+) samples and the fraction of Overall Response (OR=CR or PR) samples. Pearson correlation: cor = 0.96 (95% CI: 0.47 to 1.0), p = 0.011, alternative hypothesis: true correlation ≠ 0. The p-value is uncorrected for multiple comparisons.

f) Representative H&E micrographs for each PURE01 subtype. Scale bars are 100 μm. 'Representative' implies manual selection, with no biological or technical replicates.

g) CMap results for predicted PURE01 subtypes in the ABACUS n=84 pre-treatment cohort. CMap v1.0 connectivity score-rank distributions, with a binary heatmap showing chemical perturbagens with large negative scores.

h) Kaplan-Meier plot for DFS, in the observational (untreated) arm of the IMvigor010 n=670 MIBC cohort, for predicted subtypes S1+S4 vs. predicted subtypes S2+S3+S5. The p-value is from a log-rank test, and is uncorrected for multiple comparisons.

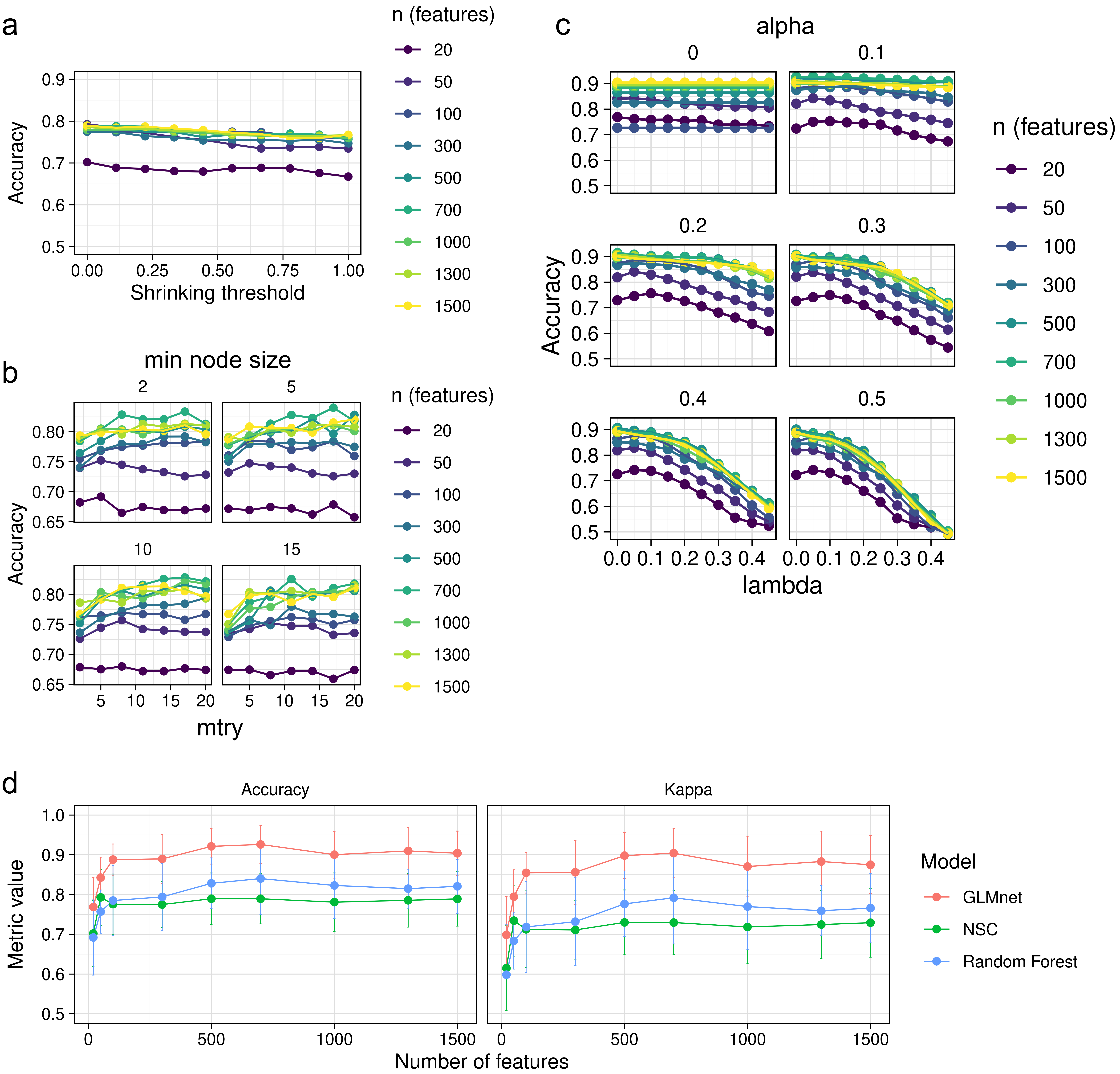

Supplementary Figure 3

Supplementary Figure 3. Parameter tuning and model accuracy for PURE01 subtype classifier models.

a) Parameter tuning for a nearest-centroid (NSC) model, showing model accuracy as a function of the shrinking threshold and the number of features.

b) Parameter tuning for a random forest (RF) model, showing model accuracy as a function of the minimum node size, mtry (the number of features to consider at each split point), and the number of features.

c) Parameter tuning for a GLMnet model, showing model accuracy as a function of parameters alpha and lambda and the number of features.

d) Accuracy and Cohen's kappa for the best GLMnet, NSC, and RF models, as a function of the number of classifier features (i.e., genes). Vertical bars indicate standard deviations of the means calculated in 3-fold repeated cross-validation, and central dots indicate the mean values. Results were generated from RNA-Seq data for the n=82 PURE01 pre-treatment cohort, with no biological or technical replicates.

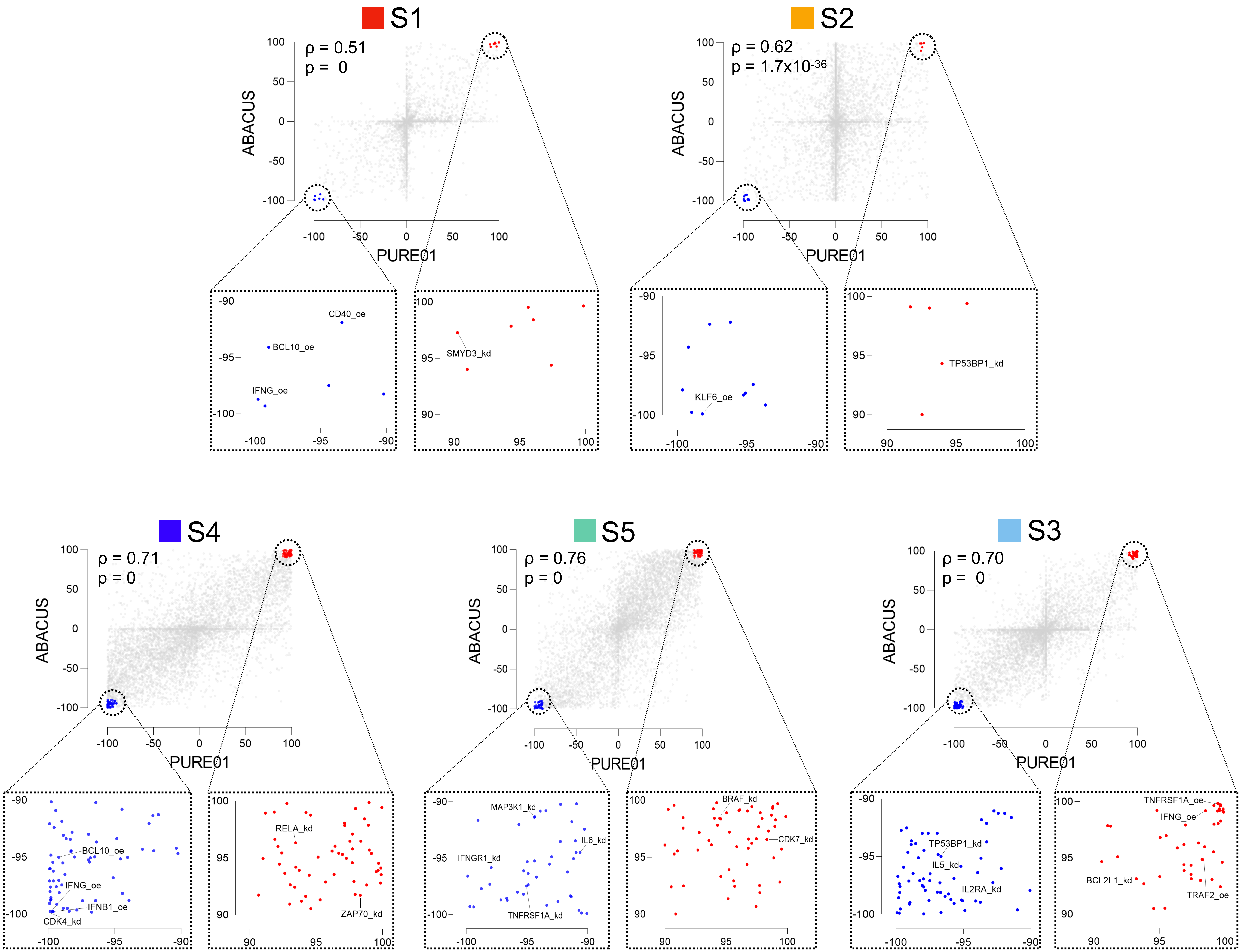

Supplementary Figure 4. Comparing CMap results for PURE01 and predicted ABACUS subtypes. Correlations between connectivity scores for perturbations in the CMap v1.0 dataset for consensus subtypes S1-5 in the PURE01 n=82 cohort and predicted subtypes S1-5 in the ABACUS n=84 cohort. For each comparison's scatterplot, we give a Spearman correlation coefficient (ρ) (alternative hypothesis = two-sided) and a *p*-value for the correlation; *p*-values are uncorrected for multiple comparisons. Correlation *rhos* were positive, indicating that the CMap expression signature for a perturbation (e.g. IFNG oe) was positively correlated with the subtype-specific PURE01 and ABACUS signature inputs. Text in each scatterplot: kd = knockdown, oe = overexpression.

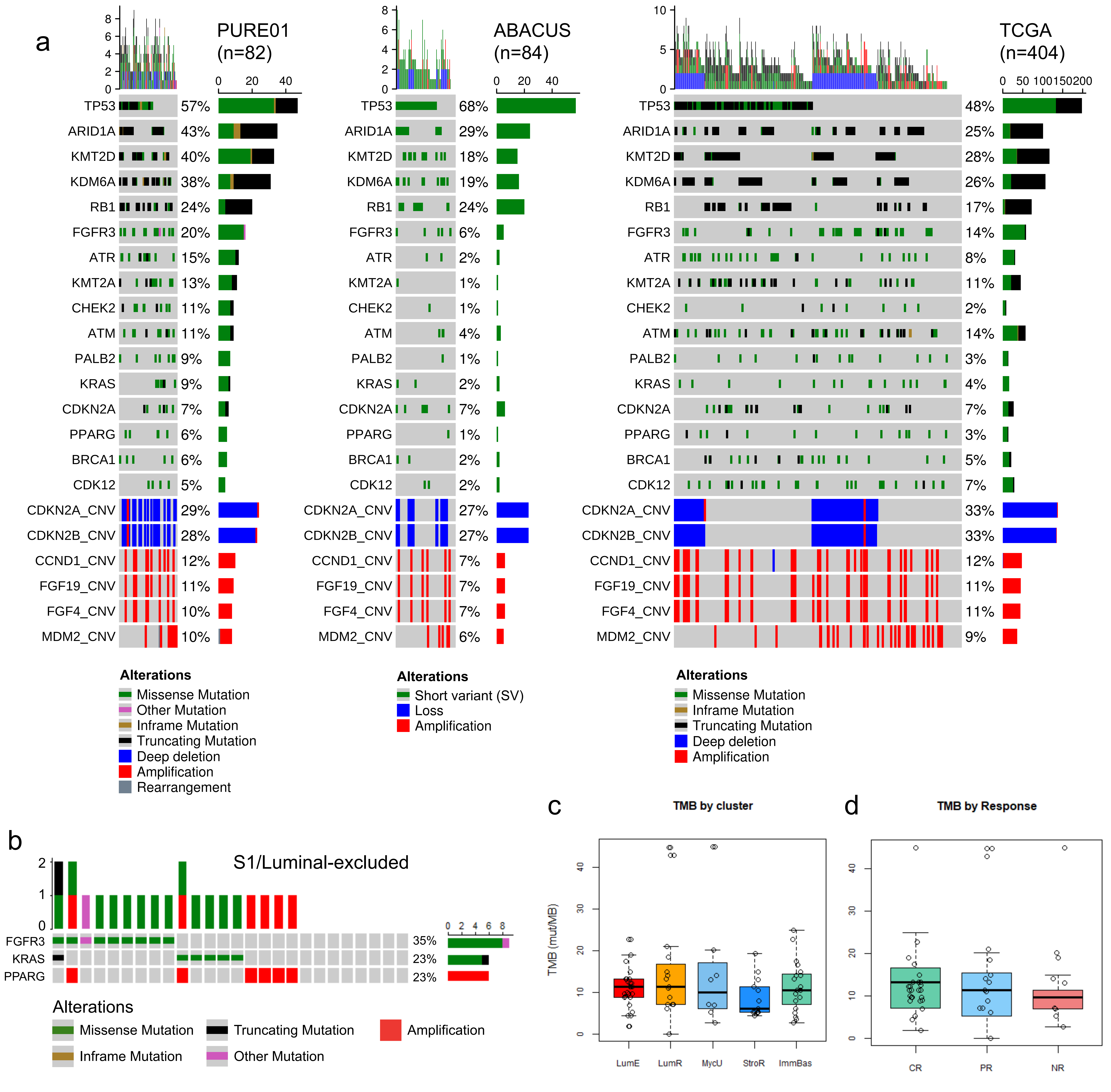

Supplementary Figure 5

Supplementary Figure 5. Somatic mutations and copy number alterations in PURE01, ABACUS, and TCGA-BLCA cohorts. Alterations in FGFR3, KRAS, and PPARG in S1. Distributions of TMB by subtype and response.

a) Oncoprints for somatic mutations, copy number alterations (CNA), and rearrangements for the PURE01 n=82 pre-treatment cohort, the ABACUS n=84 pre-treatment cohort, and the TCGA-BLCA MIBC n=404 cohort. Genes are ordered by decreasing alteration frequency in PURE01, first for mutations, then for CNA.

b) An oncoprint of FGFR3, KRAS, and PPARG alterations in the 26 samples in PURE01 subtype S1 (Luminal-excluded).

c,d) Distributions of tumor mutation burden (TMB) **c)** in PURE01 n=82 expression subtypes, and **d)** by CR, PR, and NR response in the PURE01 n=82 cohort. Boxplots are described in Methods: Statistics and reproducibility.

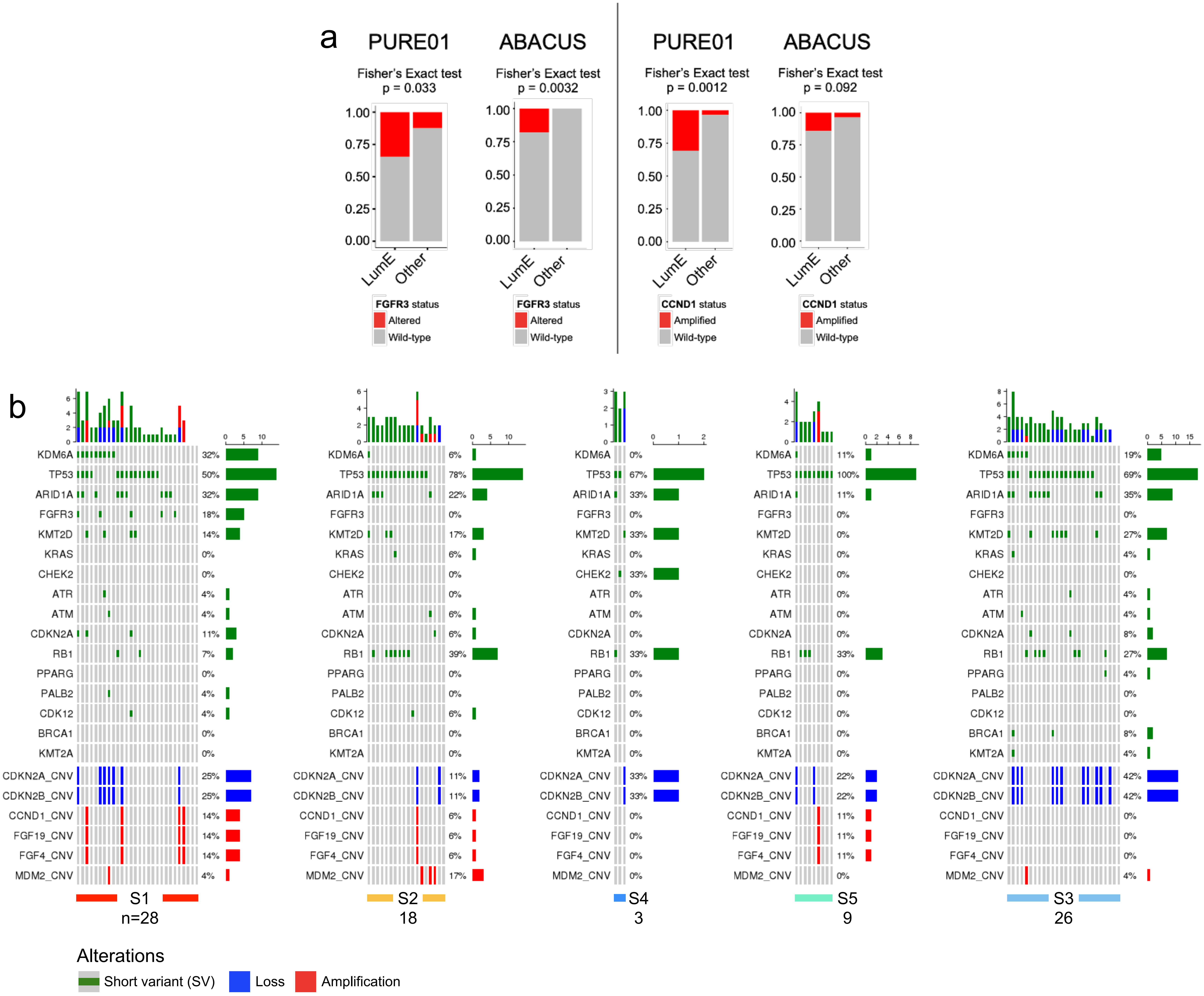

Supplementary Figure 6. Somatic alterations in PURE01 and ABACUS n=84 pre-treatment subtypes.

a) Comparing somatic FGFR3 mutations and CCND1 copy number variations between S1 and not-S1 subtypes in PURE01 and ABACUS. *P*-values are from two-sided Fisher's Exact tests, and are uncorrected for multiple hypothesis testing.

b) Somatic mutations and copy number alterations in predicted subtypes in the ABACUS n=84 pre-treatment cohort for selected genes.

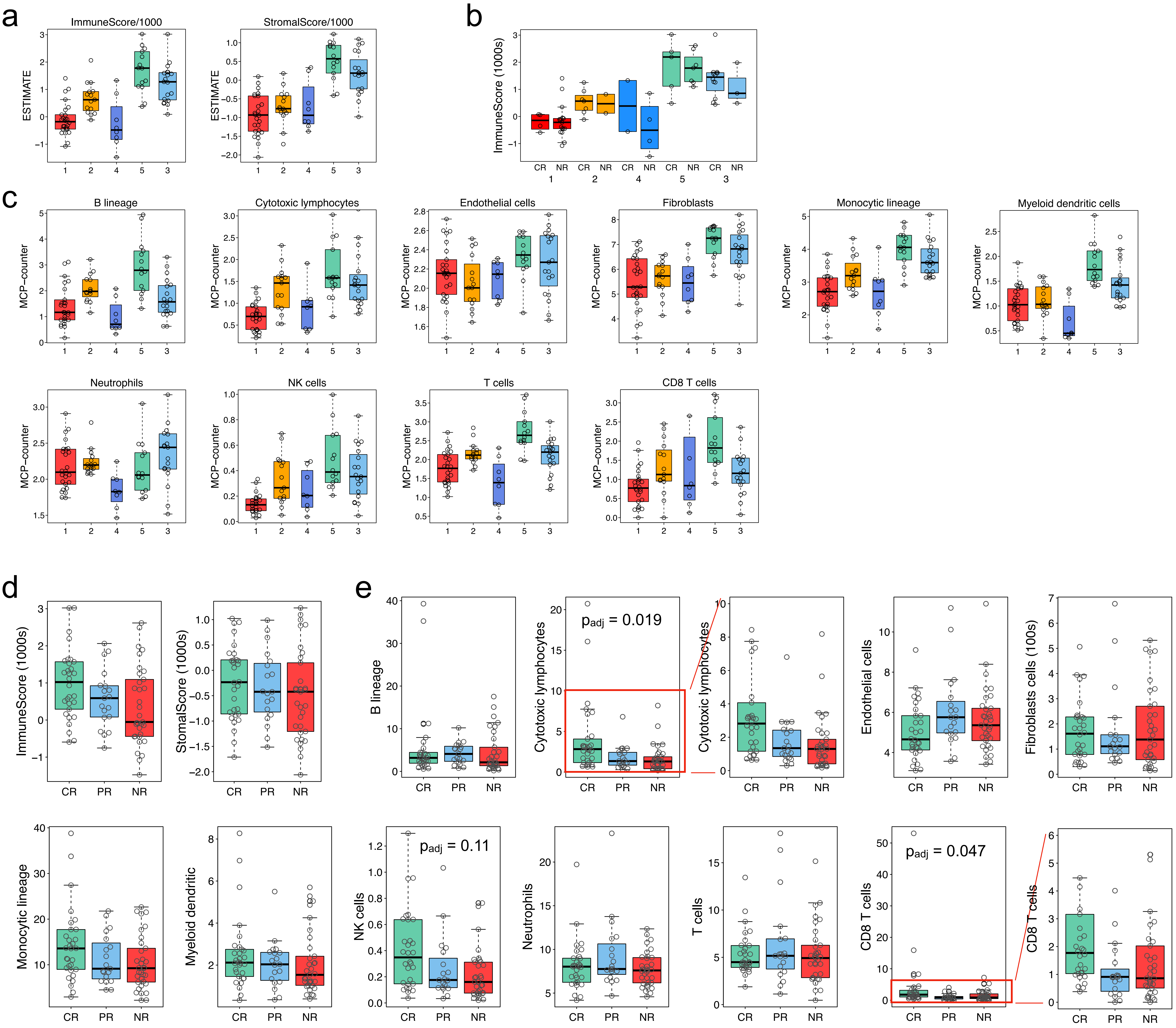

Supplementary Figure 7

Supplementary Figure 7. For five PURE01 subtypes, distributions of ESTIMATE and MCP-counter deconvolution results from bulk RNA-Seq data and distributions of protein levels from DSP data. Results were generated from RNA-Seq data for the n=82 PURE01 pre-treatment cohort, with no biological or technical replicates. Boxplots are described in Methods: Statistics and reproducibility.

a) Distributions of ESTIMATE's StromalScore and ImmuneScore in the PURE01 consensus subtypes.

b) Comparing distributions of ESTIMATE's ImmuneScore for CR and NR samples in each PURE01 subtype.

c) As (**a**), for MCP-counter's ten cell types.

d) Distributions of ESTIMATE ImmuneScore for CR, PR, and NR samples in each PURE01 subtype.

e) As (**d**), for MCP-counter's ten cell types. For Cytotoxic Lymphocytes (CTLs) and CD8 T cells, we show distributions with zoomed-in Y-axes. For CTLs, NK cells, and CD8 T cells, we report adjusted *p*-values that were calculated from two-sided Kruskal-Wallis tests, with *p*-values then Bonferroni-corrected for multiple comparisons.

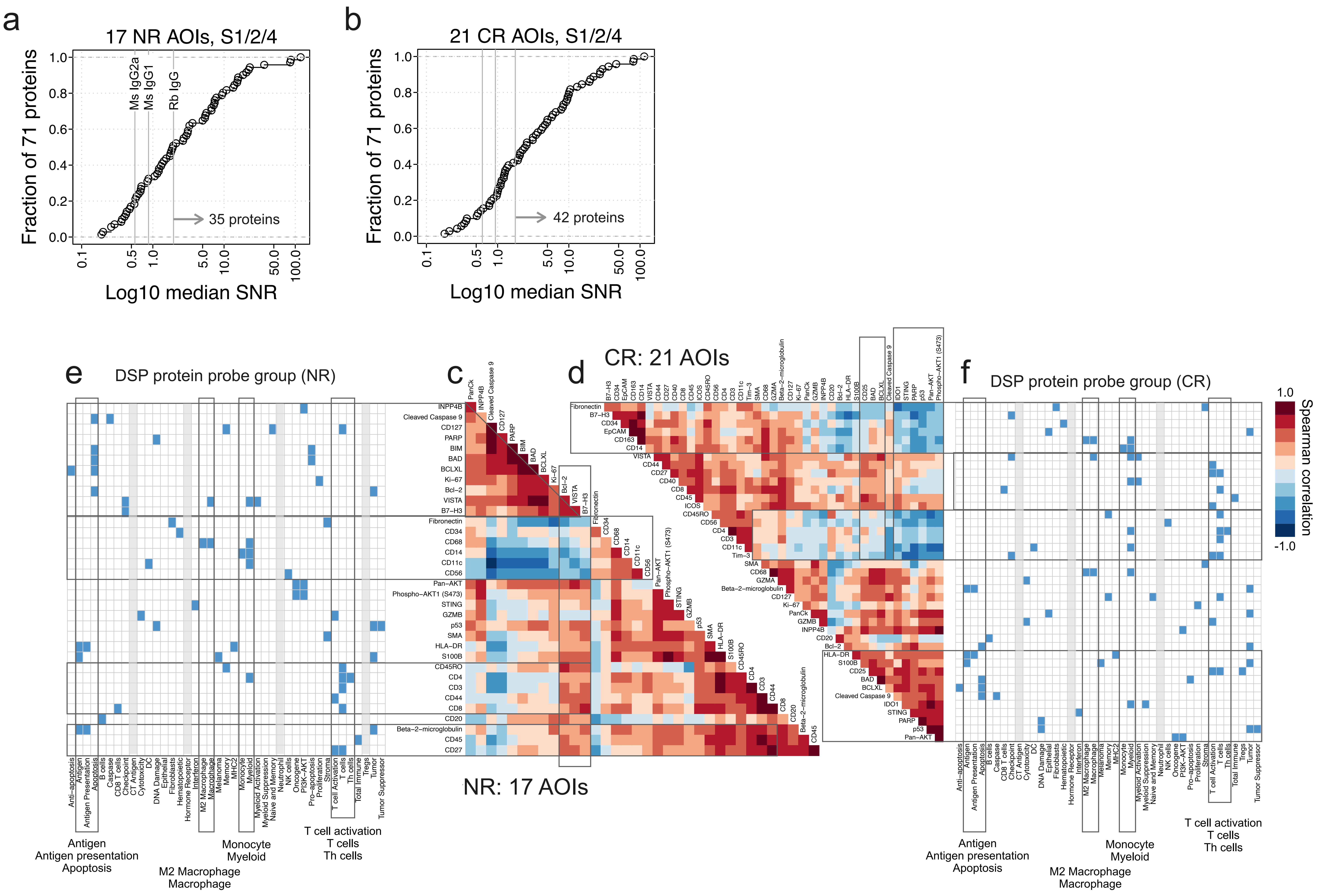

Supplementary Figure 8. DSP protein abundance and correlations for tumor microenvironment (TME) AOIs from subtypes S1, S2, and S4. See Figure 4c.

a,b) Empirical distribution functions (EDFs) of median S2N-normalized abundance for 71 proteins, and medians for three negative controls (vertical grey lines): **a)** for 17 TME AOIs from NR samples 12 (S1), 17 (S2), and 99 (S4, see Figure 4b); and **b)** for 21 TME AOIs for CR samples 88 (S1), 37 (S2), and 86 (S4). For correlations, we used only the 35 proteins that were more abundant than Rb IgG in NR samples and the 42 proteins in CR samples.

c,d) Spearman correlations for proteins with median expression above the median for the negative control Rb IgG (see **a,b**): **c)** for 35 thresholded proteins in NR samples, and **d)** for 42 thresholded proteins in CR samples. Boxes or triangles (black lines) highlight proteins with strong positive or negative correlations. Diagonal cells would have correlations of 1.0 and are not shown.

e,f) DSP functional 'probe groups' for the proteins in the correlation heatmaps.

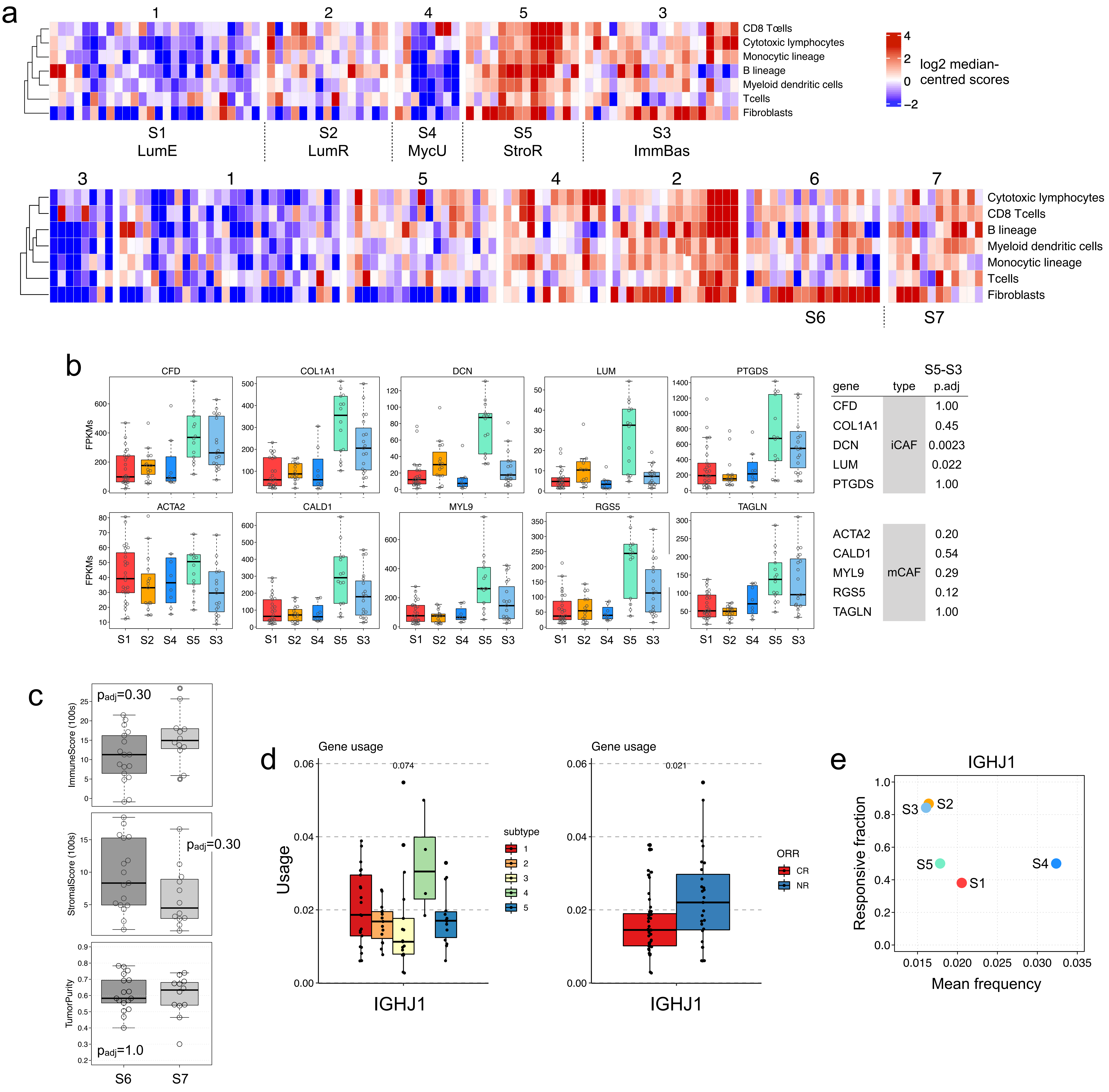

Supplementary Figure 9

Supplementary Figure 9. Immune cell types by deconvolution of bulk RNA-Seq data; expression of cancer-associated fibroblasts; and B cell receptor (BCR) gene usage. Boxplots are described in Methods: Statistics and reproducibility.

a) MCP-counter deconvolution of immune cell types for the five consensus subtypes in the PURE01 n=82 pre-treatment cohort, and the seven consensus subtypes in the PURE01 n=113 pre+post-treatment cohort. Note that the lower n=82 MCP-counter heatmap is the same as the n=82 MCP-counter heatmap in **Fig. 4a**.

b) Distributions of FPKMs for ten cancer-associated fibroblast (CAF) genes in PURE01 subtypes. The table at the right compares FPKM distributions in subtypes S5 and S3 for five iCAF and five mCAF genes. In it, we generated *p*-values with two-sided Kruskal-Wallis tests, then Bonferroni-corrected the ten *p*-values for multiple comparisons. These results were generated from bulk RNA-Seq data for the n=82 PURE01 pre-treatment cohort, with no biological or technical replicates.

c) Comparison of ImmuneScore, StromalScore, and Tumor Purity between tumors from subtypes S6 and S7. For these two groups, we compared the distributions of ESTIMATE results with Kruskal-Wallis tests, then adjusted the *p*-values for multiple comparisons with a Bonferroni correction. Results were generated from bulk RNA-Seq data for the n=82 PURE01 pre-treatment cohort, with no biological or technical replicates.

d) IGHJ1 gene usage as a function of PURE01 subtype (left), and for ORR (CR|PR) vs. NR (right). Results were generated from bulk RNA-Seq data for the n=82 PURE01 pre-treatment cohort, with no biological or technical replicates.

e) For IGHJ1, the relationship between a subtype's mean gene usage frequency and the fraction of CR samples in (CR+NR) samples.

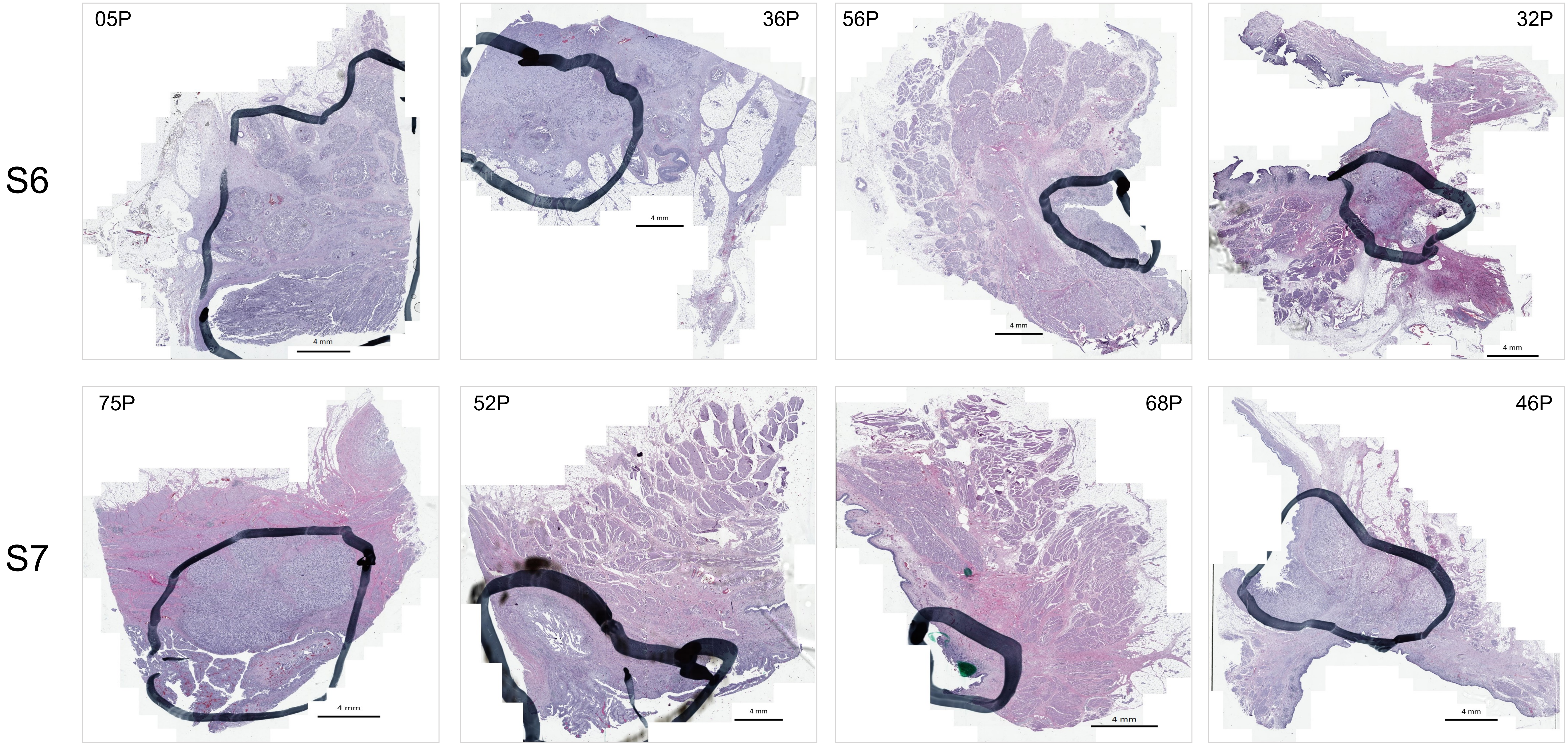

Supplementary Figure 10. Representative H&E-stained post-therapy tumors from n=113 clusters S6 and S7. The text gives sample IDs. Heavy black lines are explained in Methods. Scale bars are 4 mm.

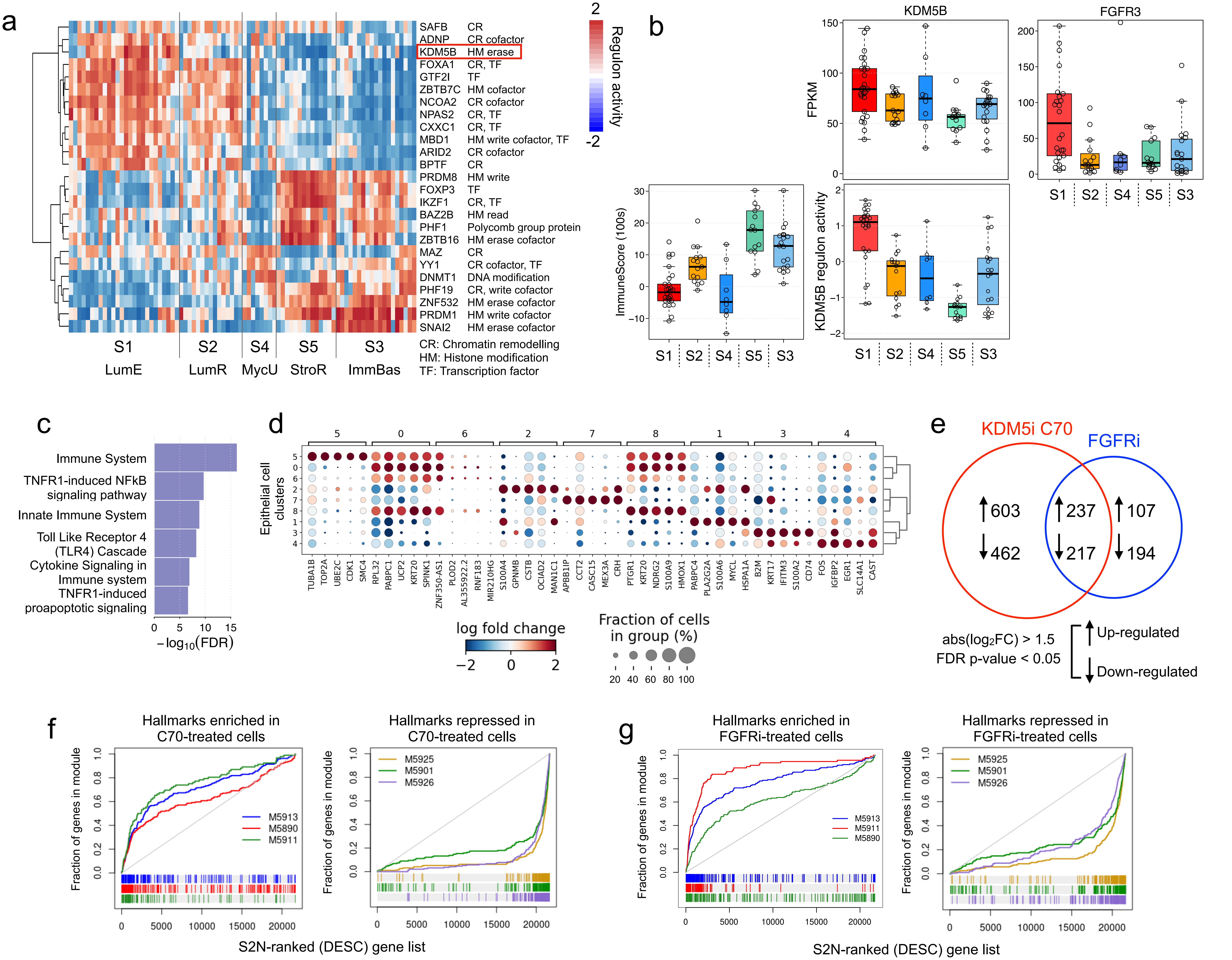

ID	Title	AUC	P.adj
M5913	IFNG response	0.71	2.4E-29
M5890	TFNA signaling via NFKB	0.63	5.8E-21
M5911	IFNA response	0.75	1.9E-20

ID	Title	AUC	P.adj
M5925	E2F targets	0.91	2.2E-123
M5926	MYC targets v1	0.91	7.0E-96
M5901	G2M checkpoint	0.82	8.6E-94

ID	Title	AUC	P.adj
M5913	IFNG response	0.77	4.6E-51
M5911	IFNA response	0.87	1.8E-41
M5890	TNFA signaling via NFKB	0.63	2.3E-17

ID	Title	AUC	P.adj
M5925	E2F targets	0.86	2.8E-80
M5901	G2M checkpoint	0.77	8.2E-60
M5926	MYC targets v1	0.79	6.0E-43

Supplementary Figure 11

Supplementary Figure 11. Regulon activities in PURE01, cell-type marker genes for scRNA-Seq data, and KDM5Bi and FGFRi treatments of RT4 cells.

a) Regulon activity heatmap across the five PURE01 n=82 subtypes for the 25 transcription factor regulons that were present in the 720 chromatin regulators in the EpiFactors DB. The text to the right gives the EpiFactors function of each regulator.

b) Above: distributions of KDM5B and FGFR3 RNA-Seq expression (FPKM) across the five PURE01 n=82 subtypes. Below: Distributions of ESTIMATE ImmuneScore and KDM5B regulon activity. Boxplots are described in Methods: Statistics and reproducibility.

c) Statistical significance of the top five Reactome pathways for the STRING-inferred interactome for the negative KDM5B regulon target genes in **Figure 6a**.

d) Expression of the top five marker genes in the nine sub-clusters identified within the epithelial cell cluster.

e) For RT4 cells, a Venn diagram comparing differentially expressed genes after treatment with the KDM5B inhibitor C70 or the FGFR inhibitor Erdafitinib. *P*-values from two-sided Fisher's Exact tests, for the overlap of significant differentially expressed genes between the two bulk RNA-Seq runs, uncorrected for multiple comparisons, were: for up-regulated genes, $p = 4.6 \times 10^{-272}$, and for down-regulated genes $p = 2.2 \times 10^{-212}$.

f,g) Enrichment plots for the top enriched and repressed MSigDB Hallmark gene sets from CERNO GSEA tests in RT4 cells treated with **f)** the KDM5i C70, and **g)** the FGFRi erdafitinib. *P*-values were calculated using CERNO tests, and were Benjamini-Hochberg- (i.e. FDR-) corrected for multiple comparisons.
