# Supplementary material for: Expression-based subtypes define pathologic response to neoadjuvant immune-checkpoint inhibitors in muscle-invasive bladder cancer
Source: Nat Commun. 2023 Apr 27;14:2126. doi: 10.1038/s41467-023-37568-9 (PMC10140274; doi:10.1038/s41467-023-37568-9)
Supplement: Supplementary file 3 — Reporting Summary [file 41467_2023_37568_MOESM3_ESM.pdf]

nature portfolio

Corresponding author(s): Joshua Meeks

Last updated by author(s): Feb 4, 2023

Reporting Summary

Nature Portfolio wishes to improve the reproducibility of the work that we publish. This form provides structure for consistency and transparency in reporting. For further information on Nature Portfolio policies, see our Editorial Policies and the Editorial Policy Checklist.

Statistics

For all statistical analyses, confirm that the following items are present in the figure legend, table legend, main text, or Methods section.

n/a	Confirmed	
☐	☒	The exact sample size (*n*) for each experimental group/condition, given as a discrete number and unit of measurement
☐	☒	A statement on whether measurements were taken from distinct samples or whether the same sample was measured repeatedly
☐	☒	The statistical test(s) used AND whether they are one- or two-sided<br>*Only common tests should be described solely by name; describe more complex techniques in the Methods section.*
☐	☒	A description of all covariates tested
☐	☒	A description of any assumptions or corrections, such as tests of normality and adjustment for multiple comparisons
☐	☒	A full description of the statistical parameters including central tendency (e.g. means) or other basic estimates (e.g. regression coefficient) AND variation (e.g. standard deviation) or associated estimates of uncertainty (e.g. confidence intervals)
☐	☒	For null hypothesis testing, the test statistic (e.g. *F*, *t*, *r*) with confidence intervals, effect sizes, degrees of freedom and *P* value noted<br>*Give P values as exact values whenever suitable.*
☒	☐	For Bayesian analysis, information on the choice of priors and Markov chain Monte Carlo settings
☒	☐	For hierarchical and complex designs, identification of the appropriate level for tests and full reporting of outcomes
☐	☒	Estimates of effect sizes (e.g. Cohen's *d*, Pearson's *r*), indicating how they were calculated

Our web collection on statistics for biologists contains articles on many of the points above.

Software and code

Policy information about availability of computer code

Data collection	clinical data for the trials (PURE01, IMVigor010 and ABACUS) have been published
Data analysis	no software was used for data collection

For manuscripts utilizing custom algorithms or software that are central to the research but not yet described in published literature, software must be made available to editors and reviewers. We strongly encourage code deposition in a community repository (e.g. GitHub). See the Nature Portfolio guidelines for submitting code & software for further information.

Data

Policy information about availability of data

All manuscripts must include a data availability statement. This statement should provide the following information, where applicable:

- Accession codes, unique identifiers, or web links for publicly available datasets
- A description of any restrictions on data availability
- For clinical datasets or third party data, please ensure that the statement adheres to our policy

Raw sequencing and variant data are deposited and available under controlled access at The European Genome-phenome Archive (EGA), which is hosted by the European Bioinformatics Institute (EBI) and the Centre for Genomic Regulation (CRG). Study accession numbers are:
(RNA-Seq data) EGAS00001004445
The remaining data are available within the Article file, Supplementary Information or from the authors upon request and included in the supp data or Zenodo

(https://doi.org/10.5281/zenodo.7750550)

Human research participants

Policy information about studies involving human research participants and Sex and Gender in Research.

Reporting on sex and gender	The clinical trial was conducted in 2016 and gender/sex were not collected separately and we are unable to report if gender and sex are not aligned. The metadata refer to gender reported by patients in the PURE01 trial.
Population characteristics	patients with bladder cancer with no prior systemic therapy of both genders
Recruitment	Recruitment for the trials were described in the original manuscripts (ref 7, 10, 24)
Ethics oversight	Expression analysis of the PURE01 cohort was reviewed by Northwestern IRB, all prior IRB approvals were reported in refs 7, 10 and 24

Note that full information on the approval of the study protocol must also be provided in the manuscript.

Field-specific reporting

Please select the one below that is the best fit for your research. If you are not sure, read the appropriate sections before making your selection.

☒ Life sciences ☐ Behavioural & social sciences ☐ Ecological, evolutionary & environmental sciences

For a reference copy of the document with all sections, see nature.com/documents/nr-reporting-summary-flat.pdf

Life sciences study design

All studies must disclose on these points even when the disclosure is negative.

Sample size	sample size was not based on power calculations but on sample availability
Data exclusions	Patients were excluded from an analysis if a tumor sample were not suitable for the specific platform (low carcinoma cell fraction, low DNA or RNA concentration, no clinical information). This resulted in the partially overlapping multi-omics analyses.
Replication	Sample replication is described in the figure legends. Replication of the classifier was performed with the Imvigor010 and ABACUS cohorts
Randomization	the trial was a single arm phase II with no randomization to other treatments
Blinding	analysis of omics data was performed independent of outcomes

Reporting for specific materials, systems and methods

We require information from authors about some types of materials, experimental systems and methods used in many studies. Here, indicate whether each material, system or method listed is relevant to your study. If you are not sure if a list item applies to your research, read the appropriate section before selecting a response.

Materials & experimental systems

n/a	Involved in the study	
☐	☒	Antibodies
☐	☒	Eukaryotic cell lines
☒	☐	Palaeontology and archaeology
☒	☐	Animals and other organisms
☐	☒	Clinical data
☒	☐	Dual use research of concern

Methods

n/a	Involved in the study	
☒	☐	ChIP-seq
☒	☐	Flow cytometry
☒	☐	MRI-based neuroimaging

Antibodies

Antibodies used	SMA (1A4; eBio 53-9760-82) 1:400<br>PanCK (AE1/AE3; Novus NBP2-33200DL594) 1:400<br>CD3 (UMAB54; Origene UM500048) 1:100

Validation	Antibodies were used at Nanostring used external antibodies, the authors did not perform validation

Eukaryotic cell lines

Policy information about cell lines and Sex and Gender in Research

Cell line source(s)	Rt4, ATCC
Authentication	cell lines were purchased commercially and not authenticated
Mycoplasma contamination	Cell lines were used after immediate purchase and not tested
Commonly misidentified lines (See ICLAC register)	no commonly misidentified cell lines were found on ICLAC

Clinical data

Policy information about clinical studies

All manuscripts should comply with the ICMJEguidelines for publication of clinical research and a completedCONSORT checklist must be included with all submissions.

Clinical trial registration	NCT02736266, NCT02662309, NCT02450331
Study protocol	described in refs 7, 10, and 24
Data collection	described in refs 7, 10 and 24
Outcomes	described in refs 7, 10, and 24
