# Supplementary material for: Expression-based subtypes define pathologic response to neoadjuvant immune-checkpoint inhibitors in muscle-invasive bladder cancer
Source: Nat Commun. 2023 Apr 27;14:2126. doi: 10.1038/s41467-023-37568-9 (PMC10140274; doi:10.1038/s41467-023-37568-9)
Supplement: Supplementary file 4 — Description of Additional Supplementary Files [file 41467_2023_37568_MOESM4_ESM.docx]

Supplementary Data Guide

Supplementary Data 1: PURE01 'master table' (includes n=82 clustering information and a subset of clinical and molecular information).

Supplementary Data 2: This work's consensus subtypes, compared to classes predicted by Lund, TCGA, cMIBC and MDA classifiers. P-values were generated with two-sided Fisher’s exact tests, and were Bonferroni-corrected for multiple comparisons.

Supplementary Data 3: Statistical association of PURE01 pathological response (CR/PR/NR) with pre-treatment subtypes. P-values were generated with two-sided Fisher’s exact tests, and were Bonferroni-corrected for multiple comparisons.

Supplementary Data 4: High-scoring Hallmark gene sets for the five PURE01 n=82 subtypes. P-values were calculated by the tmod package’s one-sided CERNO test, and were Benjamini-Hochberg-corrected for multiple comparisons, to q values (i.e. FDRs).

Supplementary Data 5: GLMnet classifier predictions for the ABACUS n=84 pre-treatment cohort

Supplementary Data 6: High-scoring Hallmark gene sets in the ABACUS n=84 cohort's five predicted PURE01 subtypes. P-values were calculated by the tmod package’s one-sided CERNO test, and were Benjamini-Hochberg-corrected for multiple comparisons, to q values (i.e. FDRs).

Supplementary Data 7: ESTIMATE results for PURE01 n=82 clusters

Supplementary Data 8: MCP-counter results for PURE01 n=82 clusters

Supplementary Data 9: PURE01: n=27 matched pre/post sample pairs

Supplementary Data 10: Consensus clusters for the PURE01 n=113 pre+post-treatment cohort

Supplementary Data 11: Matched pre-post-treatment sample pairs in n=113 consensus clusters.

Supplementary Data 12: Subtype calls for the PURE01 n=113 cohort's S6 and S7 by Lund, TCGA, consensusMIBC, and MDA classifiers

Supplementary Data 13 : Tumor areas in post-treatment PURE01 samples

Supplementary Data 14 : ESTIMATE results for PURE01 n=113 clusters

Supplementary Data 15 : MCP-counter results for PURE01 n=113 clusters

Supplementary Data 16 : Activities of 232 transcription factor regulons in the PURE01 n=82 cohort

Supplementary Data 17 : Activity status {-1,0,1} of 232 TF regulons in the PURE01 n=82 cohort

Supplementary Data 18 : Comparing KDM5B regulon targets in PURE01 n=82 and TCGA-BLCA n=404. P-values (column G) were calculated by the RTN package by permutation analysis (see Methods: Validation of the KDM5B regulon in the TCGA-BLCA cohort), and were Benjamini-Hochberg-corrected for multiple comparisons, to Adjusted P-values (FDRs, column H).

Supplementary Data 19: Enriched Reactome pathways for the STRING network for the KDM5B regulon's negative target genes

Supplementary Data 20: Probe groups for DSP proteins

Supplementary Data 21: DSP protein levels, normalized by Signal-to-noise ratio (SNR)

Supplementary Data 22: Quality control (QC) results for DSP proteins

Supplementary Data 23: DSP protein levels, normalized by housekeeping (HK) proteins

Supplementary Data 24: DSP protein levels, normalized by Scale_to_Area

Supplementary Data 25: GLMnet classifier predictions for the IMvigor010 cohort
